# Genetic and functional characterization of inherited complex chromosomal rearrangements in a family with multisystem anomalies

**DOI:** 10.1016/j.gimo.2025.103423

**Published:** 2025-03-11

**Authors:** He Fang, Stephen M. Eacker, Yu Wu, Whitney Neufeld-Kaiser, Mercy Laurino, Siobán Keel, Marshall S. Horwitz, Yajuan J. Liu

**Affiliations:** 1 Department of Laboratory Medicine and Pathology, University of Washington, Seattle WA; 2 Phase Genomics, Seattle WA; 3 Department of Genetics and Prevention, Fred Hutchinson Cancer Center, Seattle, WA; 4 Department of Medicine, Division of Hematology, University of Washington, Seattle WA

**Keywords:** Complex chromosomal rearrangements, Genomic proximity mapping (GPM), Hi-C, Optical genome mapping (OGM)

## Abstract

**Purpose:**

Complex chromosomal rearrangements (CCRs) are rare structural variants involving 3 or more chromosomal breakpoints. Most de novo-reported CCRs pose challenges for diagnosis and management. They often require karyotyping, fluorescence in situ hybridization, and chromosomal microarray analysis (CMA) for clinical diagnosis because of the limitations of each method. Here, we report an inherited, exceptionally complex CCR involving 4 chromosomes and 13 breakpoints in a family with multisystem anomalies.

**Methods:**

We evaluated the CCRs using karyotyping, fluorescence in situ hybridization, CMA, and 2 emerging genomic technologies: high-throughput chromosome conformation capture sequencing aka genomic proximity mapping and optical genome mapping. We also performed functional studies using transcriptome and methylome analyses.

**Results:**

The proband, who had intellectual disability and immune deficiency, shared CCRs with her unaffected mother involving chromosomes 1, 7, and 11 by karyotyping. However, CMA revealed a duplication and 3 deletions in the proband, in contrast to her mother’s balanced genome. High-throughput chromosome conformation capture sequencing aka genomic proximity mapping and optical genome mapping detected the CCRs and copy-number alterations but also uncovered additional breakpoints at high resolution, including an insertion in 4p and 2 cryptic rearrangements at 7p. Transcriptome and methylome analyses identified likely biological pathways associated with the proband’s phenotypes.

**Conclusion:**

Combining cytogenetic and genomic methods provided comprehensive characterization and defined the breakpoints at high resolution in both proband and mother. This underscores the value of novel cytogenetic and genomic techniques in deciphering complex genome rearrangements and the significance of integrative genomic analysis and functional characterization in understanding clinical phenotypes.

## Introduction

Constitutional complex chromosomal rearrangements (CCRs) usually involve at least 2 chromosomes and 3 breakpoints, resulting in exchanges of chromosomal segments. CCRs involving 4 chromosomes with 5 breakpoints are classified as exceptional and can be highly complex.^1^, ^2^, ^3^, ^4^ Phenotypes of people with CCRs vary, and the likelihood of a phenotypic abnormality increases with the number of breakpoints involved in apparently balanced CCRs.^3^^,^^5^^,^^6^ Abnormal phenotypes from CCRs can result from disruption of dosage-sensitive genes, cryptic genomic imbalances near the breakpoints, alteration of the expression of disease-candidate genes, and unmasking of recessive variants on the intact chromosome.^7^, ^8^, ^9^, ^10^ Most familial transmission of CCRs is through the mother.^2^^,^^11^

Precise characterization of CCRs is crucial but challenging with conventional cytogenetics tools. Traditional cytogenetic tools face significant limitations when characterizing CCRs. Although karyotyping provides a whole-genome view at the single-cell level, its resolution is limited, making it challenging to detect smaller rearrangements. Fluorescence in situ hybridization (FISH) can identify specific rearrangements with high precision but is restricted in scope because it only targets known regions. Single-nucleotide polymorphism (SNP) chromosomal microarray analysis (CMA) offers high-resolution detection of copy-number variants (CNVs) and regions of homozygosity; yet, it cannot identify balanced rearrangements, such as translocations, inversions, or other CCRs that do not involve copy-number changes. For example, a balanced translocation that disrupts a critical gene would go undetected by CMA, highlighting the need for more comprehensive approaches in CCR analysis

High-throughput chromosome conformation capture sequencing (Hi-C; aka genomic proximity mapping (GPM)) and optical genome mapping (OGM) are 2 technologies that capture ultralong-range contiguity information and detect all types of structural variants (SVs) in a single assay. OGM is an imaging-based method that produces DNA fingerprints spanning very large genomic regions.^12^ GPM (Hi-C) is a chromatin conformation analysis that identifies chromatin contacts within the nucleus by proximity ligation followed by next-generation sequencing.^13^ We evaluate the performance of GPM (Hi-C) and OGM in clinical settings with a family with complex CCRs and show that both methods, although based on different principles, are powerful tools to precisely define the breakpoints of inherited CCRs involving chromosomes 1, 4, 7, and 11.

CCRs may exert a pathogenic effect by gene dosage-dependent mechanisms or through disruption of the genomic architecture that predominantly affect gene expression.^10^^,^^14^, ^15^, ^16^ Such inferred mechanisms of pathogenicity need corroboration by messenger RNA (mRNA) sequencing. CCRs can also reshape epigenetic landscapes, including DNA methylation, chromatin structure, and posttranslational modifications leading to disease. We hypothesized that investigating transcriptomic and epigenetic landscapes could explain the pathogenic consequences of the CCRs in this family.

## Materials and Methods

### Proband consents

Written informed consent was obtained from the proband and the proband’s mother. They were enrolled in this study under a protocol approved by the Institutional Review Board. Peripheral blood samples were obtained for DNA and RNA studies.

### Cytogenetics and SNP CMA

Karyotyping was performed on phytohemagglutinin-stimulated peripheral blood lymphocytes according to standard procedures. Twenty GTG-banded metaphases were analyzed. Metaphase FISH analysis was performed on cultured peripheral blood cells using subtelomere probes for chromosomes 1p, 1q, 7p, 7q, 11p, and 11q. CMA of genomic DNA prepared from peripheral blood was performed using the Illumina Infinium CytoSNP-850K BeadChip v1.1. Microarray data were visualized and analyzed using Illumina BlueFuse Multi v4.4 (Illumina, Inc) and NxClinical version 10.0 (Biodiscovery, Inc). All analyses and genomic coordinates reported in this manuscript are based on the human genome build GRCh38/hg38.

### GPM(Hi-C)

GPM(Hi-C) libraries were generated using the Phase Genomics Proximo Human kit v4.0 (Phase Genomics Inc) following the manufacturer’s protocol. In brief, white cell pellets from the peripheral blood samples were crosslinked, preserving the chromatin structure within the intact nucleus. After cell lysis, chromatin was immobilized on magnetic beads and digested using restriction enzymes. The overhangs were filled in with biotinylated nucleotides and subjected to proximity ligation. Ligated junctions were purified using streptavidin beads and converted to a standard dual-indexed Illumina-compatible library. In total, we sequenced approximately 200 million Hi-C reads for each of the proband and her mother. Paired-end sequencing data were processed using the Juicer pipeline. The frequency of spatial contact is represented with heatmaps. Hi-C matrices were corrected afterward using “hic_CorrectMatrix” tools from HiCExplorer v1.8.1. Topologically associating domain (TAD) boundaries were called using the “hicFindTADs” tool from HiCExplorer v1.8.1. First eigenvector (PC1) corresponding to active (A) and inactive (B) compartments was computed using “hicPCA -noe1 –norm” from HiCExplorer after the removal of heterochromatic chromosome ends. The correct orientation of PC1, that is, positive values corresponding to the active compartment (A) and negative values corresponding to the inactive compartment (B), was verified for each chromosome using publicly available ENCODE ChIP-seq data.

Hi-C matrices were visually inspected to evaluate the presence of SVs. The Hi-C matrix is a heatmap representation of pairwise sequence interaction frequency across the genome. Sequences that are physically close together on a chromosome interact more frequently that sequences that are more distant. An SV can be detected on the heatmap as a deviation in the expected pattern of pairwise sequence interactions. For instance, a translocation appears as an excess of interchromosomal interaction between 2 chromosomes participating in the rearrangement.^13^^,^^17^ The breakpoint can be identified as the region of the heatmap with highest levels of interchromosomal interaction, with the intensity of signal decaying following a power law function as it extends away from the breakpoints. Interchromosomal interactions typically demonstrate sharp boundaries of signal interaction that correspond with the breakpoint boundaries. Intrachromosomal SVs can also be detected using similar principles but, instead, by observing deviations in expected intrachromosomal sequence interactions. Again, breakpoints can be identified by observing the highest level of unexpected pairwise sequence interactions and the power law decay of these interactions as they extend from the breakpoint. CNVs can be detected from Hi-C sequencing data using traditional sequence coverage and allele-frequency based methods, and in this study were evaluated using NxClinical version 10.0 (Biodiscovery, Inc). Sequence data generated as a part of this study were also analyzed using the CytoTerra GPM cloud analysis platform (Phase Genomics, Inc). Using a suite of automated variant calling algorithms, CytoTerra identified the variants described in this study, providing independent support for the manual interpretations presented here. All analyses and genomic coordinates reported in this manuscript are based on the human genome build GRC38/hg38.

### OGM

Ultrahigh molecular weight DNA was extracted from white blood cells and labeled following the manufacturer’s protocols (Bionano Genomics). The fluorescently labeled DNA molecules were loaded on flow cells and imaged sequentially across nanochannels on a Saphyr instrument. A median coverage of >100x was achieved for both samples. The proprietary OGM-specific software—Bionano Access and Solve (versions 1.6/1.7 and 3.6/3.7, respectively), were used for data processing. De novo assembly was performed using Bionano’s custom assembler software program based on the Overlap-Layout-Consensus paradigm. SVs were identified based on the alignment profiles between the de novo assembled genome maps and the Human Genome Reference Consortium GRCh38/hg38 assembly. Fractional copy-number analyses were performed from alignment of molecules and labels against GRCh38/hg38.

### mRNA sequencing

Library preparation for RNA-sequencing (RNA-seq) was performed on 100 ng of total RNA isolated from peripheral blood using TruSeq Stranded mRNA Sample preparation kit (Illumina, Inc). The library’s size distribution was validated, and quality inspected on a Bioanalyzer TapeStation (Agilent Technologies). High-quality libraries are pooled for 75-bp paired-end sequencing on a NextSeq500 instrument (2 × 75 cycles) according to manufacturer instructions (Illumina, Inc).

Reads were aligned to the human annotation reference genome GRCh38 using STAR 2.5.2b. BAM files of mapped reads were visualized by the integrative genomics viewer. A total of 62 and 71 million (M) uniquely mapped reads (mappability > 84%) were obtained for the proband and proband’s mother, respectively. RNA-seq data were compared with public mRNA-sequencing (mRNA-seq) data sets from whole blood of 6 females age matched to the proband and proband’s mother (RNA-seq whole blood of Dutch 500FG cohort, National Library of Medicine National Center for Biotechnology Information, Gene Expression Omnibus, GSE134080). Libraries for the external controls were prepared using TruSeq mRNA Sample prep kit. The single-end read-length is 100 bp. These control data sets have a similar sequence depth and quality, with approximately 25 million uniquely mapped reads (mappability > 87%), which were reprocessed with our own data for normalization and comparison.

Differential expression (DE) profile analyses were done with weighted trimmed mean of M-values normalization method and GENCODE gene annotation in the EdgeR statistical software package (Bioconductor) to investigate the relative change in gene expression (ie, normalized counts) between different samples. Two DE comparisons were conducted: (1) the proband versus 3 healthy age-matched female controls and (2) proband’s mother versus 3 healthy age-matched female controls. A total of 11,460 expressed genes with a median of over 5 raw counts per million for the 8 samples (the proband, proband’s mother and 6 control females) were included for DE analyses. Absolute expression fold changes of 2 and false discovery rate (FDR) < 0.01 were set as the threshold to call genes with significantly differentially expressed genes (DEGs; Supplemental Table 1). DEGs were used for Gene Ontology (GO) enrichment analysis.

GO overrepresentation test was done by PANTHER to test whether upregulated or downregulated DE genes are enriched in GO terms (eg, biological processes and pathways) compared with the reference of 20,996 human genes. FDR < 0.05 from Fisher exact test was used for the cutoff. Biological processes or pathways with enrichment fold < 1 were not shown.

### Methylation array data processing

We performed methylome analysis with the proband’s peripheral blood samples and an age-matched normal female control using Illumina Infinium Human Methylation EPIC BeadChip (Illumina, Inc). This methylation array covers over 850,000 methylation sites across the genome at single-nucleotide resolution. DNA methylation profiling was performed according to the manufacturer’s instructions. We performed differential DNA methylation analysis using a customized R package pipeline. We stratified quantile normalized data using the “minfi” “preprocessQuantile” function. After this, probes targeting sex chromosomes, containing SNPs, not uniquely matching, and known cross-reactive probes were removed. Most significantly, differentially methylated CpG were identified by fitting a regression model with the disease as the target variable using the “limma” R package. Differentially methylated gene lists identified through methylation array analysis were used for GO enrichment analysis. GO overrepresentation test was done by PANTHER to test whether upregulated or downregulated DE genes are enriched in GO terms (eg, biological processes and pathways) compared with the reference of 20,996 human genes. FDR < 0.05 from Fisher exact test was used for the cutoff. Biological processes or pathways with enrichment fold < 1 are not shown.

## Results

### Clinical presentation

The proband is a 31-year-old female with a history of systemic lupus erythematosus (SLE) with Sjogren syndrome and suspected lupus nephritis, immune thrombocytopenic purpura (ITP), reactive lymphadenopathy, and developmental delay. She was the product of a term pregnancy to a then 28-year-old mother, delivered by Cesarean section for breech presentation. Maternal exposures were limited to low-dose aspirin prescribed for recurrent pregnancy loss. The proband’s early developmental milestones were normal except for delays in fine motor skills, toilet training and expressive language. She was diagnosed with developmental delay at age 7 years, without a specific recognizable syndrome, based on Wechsler Intelligence Scale testing. She completed high school through a special education program, remains independent in activities of daily living, reads, watches television, and lives with others in an adult family home.

The proband’s medical history is notable for her recurrent urinary tract infections as a child secondary to urinary incontinence and attention-deficit/hyperactivity disorder. She underwent tonsillectomy for recurrent pharyngitis at the age of 9 years and was treated as an outpatient for pneumonia twice during childhood. At approximately age 25, the proband developed reactive cervical, paraaortic, and inguinal lymphadenopathy. Cervical lymph node excisional biopsy revealed follicular hyperplasia without immunophenotypic evidence for lymphoma. Workups for infectious etiologies, including HIV and HHV8 serologies, were negative, and serum protein electrophoresis revealed polyclonal hypergammaglobulinemia. Around the same time, as part of an evaluation for keratoconjunctivitis sicca, along with nonnephrotic-range proteinuria, the proband was diagnosed with SLE and Sjogren syndrome based on positive antineutrophil antibody, antidouble-stranded DNA, Sjögren's syndrome antigens A (anti-SSA), Sjögren's syndrome antigens B (anti-SSB) testing, and low C4. The proband’s keratoconjunctivitis sicca improved and lymphadenopathy resolved while on hydroxychloroquine therapy. At age 31, she presented with bruising and was found to have isolated severe thrombocytopenia (platelet count: 5000/μL). Her platelet count normalized with intravenous immunoglobulin and prednisone therapy, consistent with a diagnosis of ITP. The proband has since received mycophenolate mofetil as a steroid-sparing agent for ITP, and the noted proteinuria also resolved during the course of therapy with mycophenolate mofetil.

Physical examination revealed a pleasant, appropriately verbally communicative female weighing 104 kg with a height of 1.7 m. She had subtle midface hypoplasia with epicanthic folds, hypertelorism, an appearance of a webbed neck, and triangular-shaped fingers. The proband’s mother is a healthy, 60-year-old female with a history of 2 miscarriages with a partner different from the proband’s father. She completed high school, has been employed as a licensed practical nurse, and has an unremarkable medical history with no known autoimmune or other disorders. Her physical examination was unremarkable.

### Cytogenetics and SNP CMA results

**Figure 1:**
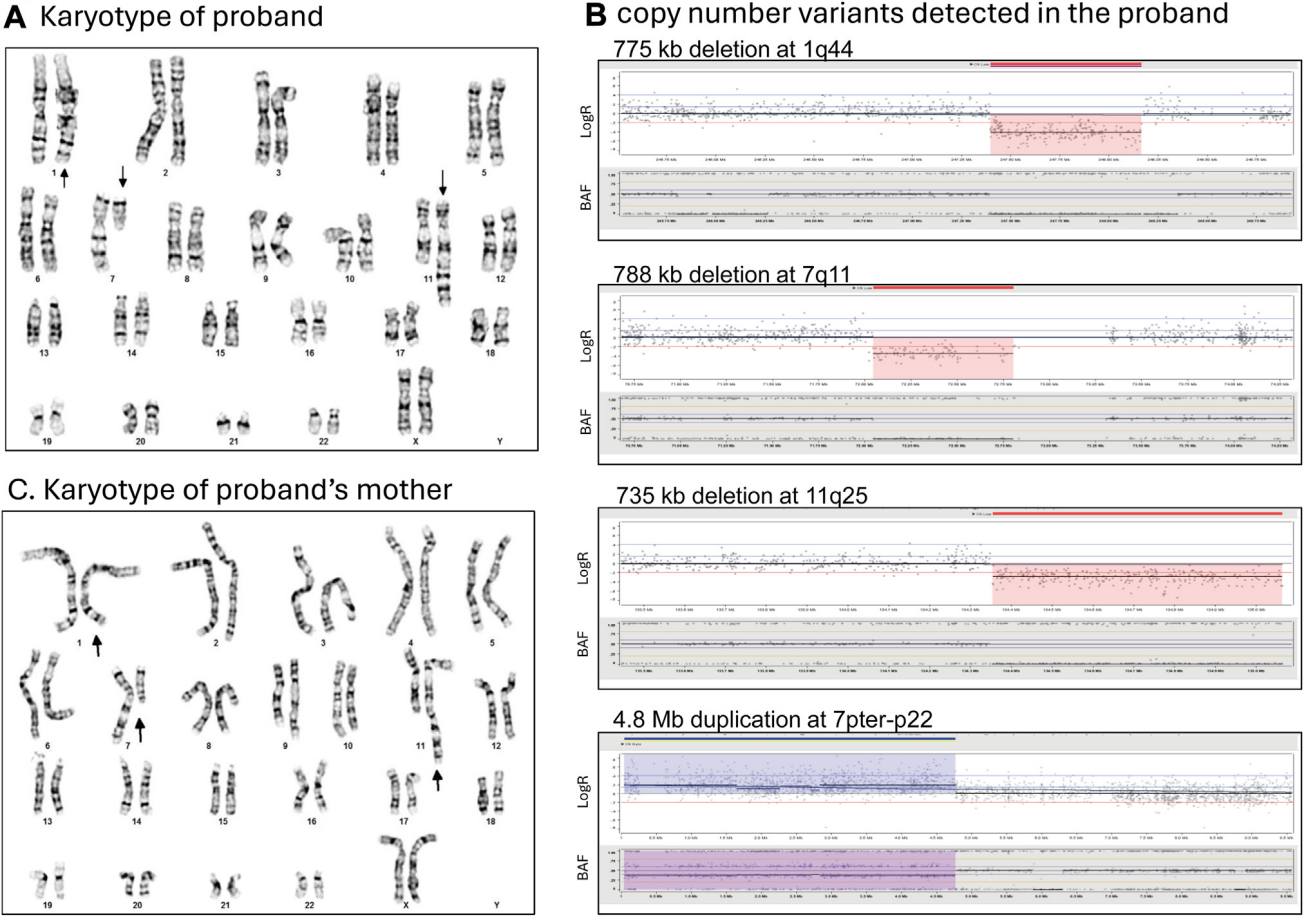
**Results of G-banded chromosome analysis and single-nucleotide polymorphism (SNP) genomic microarray analysis of the proband and the proband’s mother.** A. Karyotype of the proband. B. Four copy-number variants detected in the proband by SNP genomic microarray analysis with genomic coordinates on the *x*-axis and Log_2_R and B-allele frequency (BAF) on the *y*-axis (see Table 1 for details). C. Karyotype of the proband's mother. The arrows in (A) and (C) point out the abnormal chromosomes 1, 7, and 11 in the proband and the proband’s mother, respectively.

The proband’s karyotype analysis revealed a complex but seemingly balanced set of rearrangements: 46,XX,der(1)t(1;7)(q42.2;p22),der(7)t(1;7)(q44;q11.2),der(11)(pter->11q25::1q42->1q44::7q11.2->7qter) (Figure 1A). This exceptional CCR involves translocations of chromosomes 1, 7, and 11, with breakpoints at 1q42.2, 1q44, 7p22, 7q11.2, and 11q25, respectively.

To determine whether the CCR was truly balanced, we performed genome-wide CMA. This revealed 4 CNVs: a 4.8-Mb terminal duplication of 7pter-p22 and 3 deletions of 774 kb, 788 kb, and 675 kb from 1q44, 7q11, and 11q25-qter, respectively (Figure 1B, Table 1). The CNVs do not overlap any known dosage sensitive genes or regions, and the classification of each of these regions is uncertain per American College of Medical Genetics and Genomics (ACMG) interpretation standards. None of the CNVs were inherently pathogenic. The 7q11 deletion does not overlap the Williams syndrome critical region; the 11q25-qter deletion does not contain the critical region for Jacobsen syndrome, and the 1q44 deletion does not encompass the critical region for 1q43q44 microdeletion syndrome.

**Table 1:** Copy number variations (CNVs) detected by SNP CMA, GPM (Hi-C), and OGM in the proband

CNVs	CMA	GPM (Hi-C)	OGM
Loss on Chr1	arr[GRCh38] 1q44(247396871_248171504)x1	gpm[GRCh38] 1q44(247415001_248170001)x1	ogm[GRCh38] 1q44(247286043_248164170)x1
Gain on Chr7	arr[GRCh38] 7p22.3p22.1(1_4799433)x3	gpm[GRCh38] 7p22.3p22.1(1_4785001)x3	ogm[GRCh38] 7p22.3p22.1(706747_4738703)x3
Loss on Chr7	arr[GRCh38] 7q11.22q11.23(72046323_72834567)x1	gpm[GRCh38] 7q11.22q11.23(72055001_72840001)x1	ogm[GRCh38] 7q11.22q11.23(71995945_72863337)x1
Loss on Chr11	arr[GRCh38] 11q25(134351449_135086622)x1	gpm[GRCh38] 11q25(134350001_135070000)x1	ogm[GRCh38] 11q25(134309117_135069566)x1

*CMA*, chromosomal microarray analysis; *GPM (Hi-C)*, genomic proximity mapping (GPM) (aka high-throughput chromosome conformation capture sequencing [Hi-C]); *OGM*, optical genome mapping; *SNP*, single-nucleotide polymorphism.

The detection of CNVs in the proband showed that the proband’s CCR was actually unbalanced. The terminal duplication at 7pter-p22 likely encompasses the breakpoints on the derivative chromosome 1 as identified by karyotype. Similarly, the deletions at 1q44, 7q11 and 11q25-qter likely encompass the breakpoints on the derivative chromosomes 7 and 11. To determine if the proband’s CCR was inherited or de novo, a peripheral blood sample from the proband’s mother was requested for karyotyping and CMA. The proband’s father was not available for testing. Surprisingly, the proband’s mother’s karyotype seemed indistinguishable from the proband, with complex rearrangements involving chromosome 1, 7, and 11 (Figure 1C). In contrast, the proband’s mother’s CMA revealed no reportable CNVs per ACMG interpretation standards (Supplemental Figure 1). This finding confirms that the rearrangement in the mother is truly balanced, which aligns with her unremarkable clinical presentation. This result also consistent with the mother’s history of 2 miscarriages, suggesting that the balanced nature of her rearrangement allowed for normal health but may have contributed to reproductive challenges. Furthermore, this result highlights the limitation of karyotyping in detecting cryptic rearrangements, particularly in subtelomeric regions, where its resolution (3-10 Mb) is insufficient.

**Figure 2:**
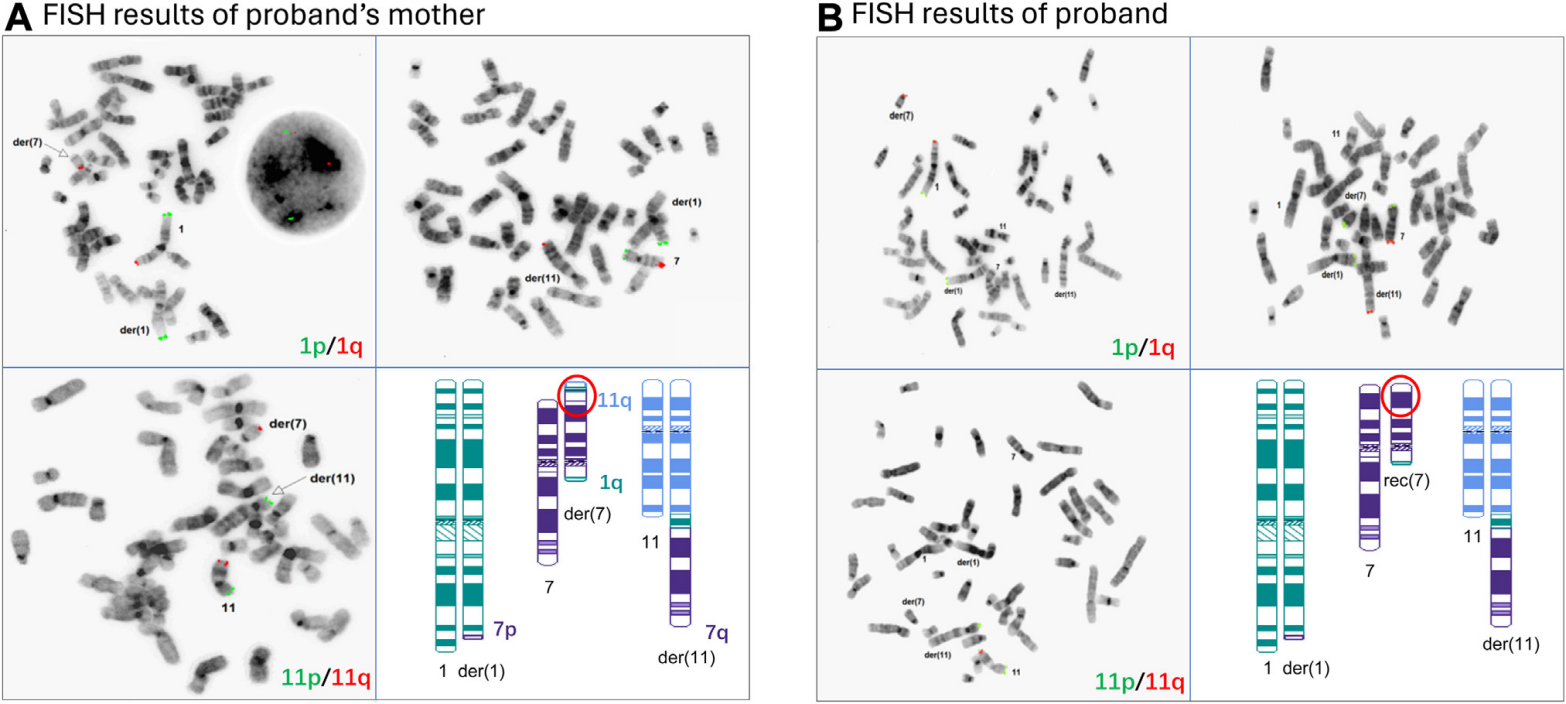
**Fluorescence in situ hybridization (FISH) analysis results of the proband’s mother (A) and the proband (B).** Dual-color FISH using subtelomeric probes set on metaphase cells identified localizations of 1p (green) and 1q (red), 7p (green) and 7q (red), 11p (green) and 11q (red) on chromosome 1, der(1), chromosome 7, der(7), chromosome 11, and der(11). Schematic diagrams in the lower right corner of (A) and (B) show localization of FISH signals on ideograms of the respective rearranged chromosomes.

Metaphase FISH was performed using subtelomeric probes that hybridize to 1pter, 1qter, 7pter, 7qter, 11pter, and 11qter (Figure 2). Consistent with the proband’s mother’s normal CMA results, 2 signals each were seen for 1qter on chromosome 1 and derivative chromosome 7, 7pter on chromosome 7 and derivative chromosome 1, and 11qter on chromosome 11 and derivative chromosome 7 (Figure 2A). In contrast, the proband had 2 signals of 1qter on chromosome 1 and derivative chromosome 7, but 3 signals of 7pter on chromosome 7, derivative chromosome 7, and derivative chromosome 1 and 1 signal of 11qter on chromosome 11 (not on derivative chromosome 7, as in her mother) (Figure 2B). The FISH results confirmed the unbalanced nature of the CCR in the proband and was consistent with the CMA results.

### CCR breakpoint mapping by GPM (Hi-C) and OGM

**Figure 3:**
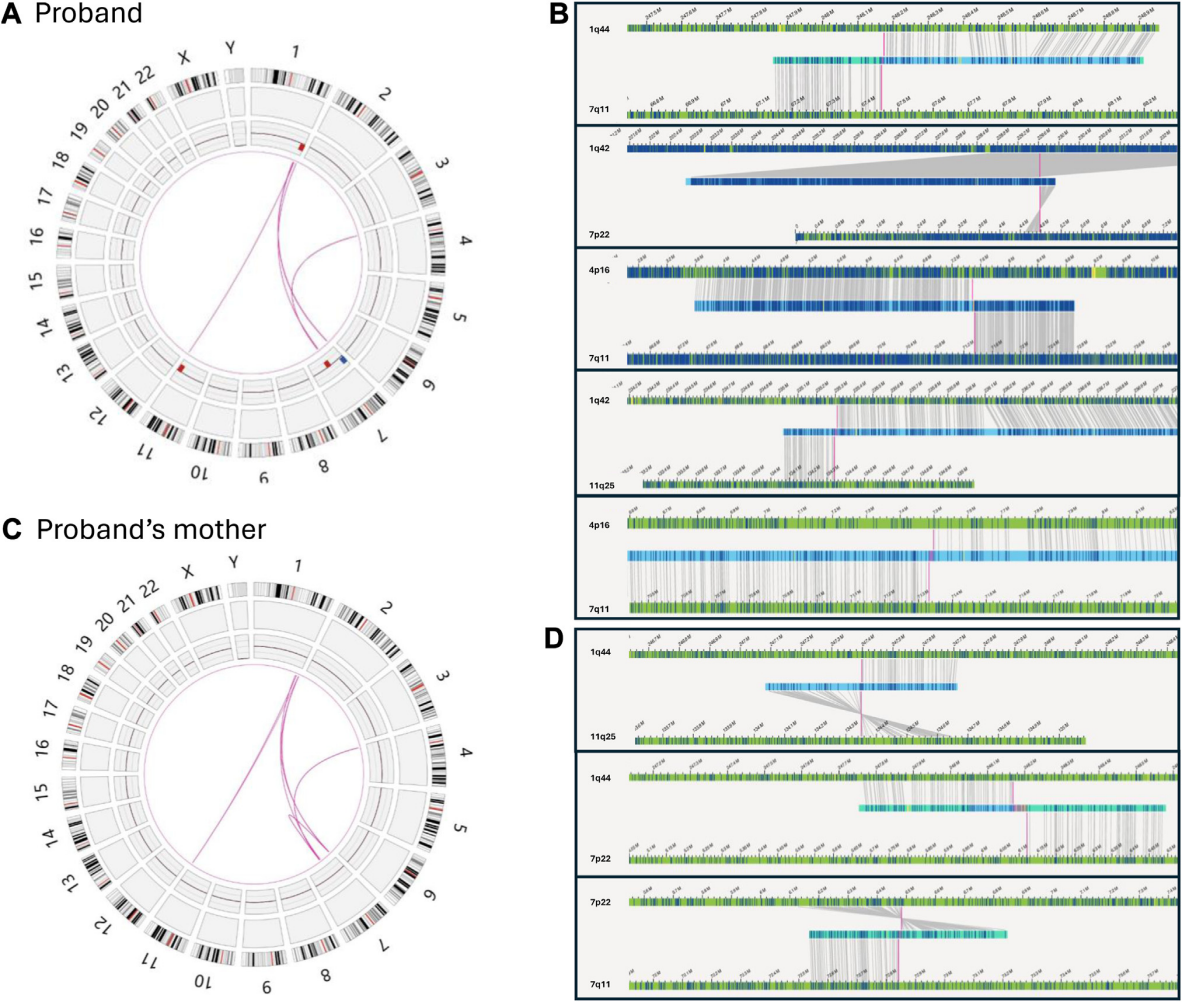
**Optical genome mapping results in the proband and her mother.** Circos plots display the complex chromosomal rearrangements (CCRs) in the proband (A) and the proband’s mother (C). The outer rings represent chromosomes 1-22, X and Y; inner rings show the CCRs detected. Circos tracks include cytoband, structural variant track, copy-number variant track (red deletion, blue duplication) and translocations. (B and D) Detailed views of rearrangements around the breakpoints. In the proband, a total of 5 translocations were shown (B). In the proband’s mother, 8 translocations observed, including 5 same as those in the proband (C) and 3 in the proband’s mother only (D).

GPM (Hi-C) and OGM were performed to precisely characterize the breakpoints of both unbalanced and balanced CCRs in the proband and her mother. OGM simultaneously identified all 4 CNVs (Table 1) and the SVs involving chromosomes 1, 7, and 11 in the proband (Figure 3A). The breakpoints detected by OGM were 1q42, 1q44, 7p22, 7q11.2, and 11q25, consistent with the karyotype (Figure 3B). OGM also clarified the breakpoints at the gene level, indicating disruption of *ARID4B* (HGNC:15550), *GLB1L2* (HGNC:25129), *URB2* (HGNC:28967), and *AP5Z1* (HGNC:22197) in the proband (Table 2). These rearrangements correspond to the aberrations we observed on der(1), der(7), and der(7) via karyotype. Moreover, a novel balanced insertion between chromosome 4 and chromosome 7, which was not observed by any conventional cytogenetic test method, was identified (Figure 3A and B).

**Table 2:** Breakpoint coordinates of structural variants (SV) detected by GPM (Hi-C) and OGM in the proband and proband’s mother

Case	SV	Translocations Detected by GPM (Hi-C) [hg38]	Translocations Detected by OGM [hg38]
Proband	t(1;11)	gpm[GRCh38] t(1;11)(q42.3;q36)(235305001;134380001)	ogm[GRCh38] t(1;11)(q42.3;q25)(235309987;134339794)
	t(4;7)	gpm[GRCh38] t(4;7) (p16.1;q11.22)(7484910;71335001)	ogm[GRCh38] t(4;7)(p16.1;q11.22)(7484910;71350922)
	t(4;7)	gpm[GRCh38] t(4;7) (p16.1;q11.22)(7484910;71335001)	ogm[GRCh38] t(4;7)(p16.1;q11.22)(7498374;71330734)
	t(1;7)	gpm[GRCh38] t(1;7) (q42.13;p22.1)(229625001;4780001)	ogm[GRCh38] t(1;7)(q42.13;p22.1)(229627661;4784641)
	t(1;7)	gpm[GRCh38] t(1;7) (q44;q11.21)(248170001;67460001)	ogm[GRCh38] t(1;7)(q44;q11.21)(248174298;67453424)
Proband’s mother	t(1;11)	gpm[GRCh38] t(1;11)(q42.3;q41)(235305001;134380001)	ogm[GRCh38] t(1;11)(q42.3;q27)(235309987;134339794)
	t(1;11)	gpm[GRCh38] t(1;11)(q44;q25)(247390001;134345001)	ogm[GRCh38] t(1;11)(q44;q25)(247397537;134350029)
	t(4;7)	gpm[GRCh38] t(4;7)(p16.1;q11.22)(7485001;71351001)	ogm[GRCh38] t(4;7)(p16.1;q11.22)(7484910;71350922)
	t(4;7)	gpm[GRCh38] t(4;7)(p16.1;q11.22)(7498401;71321501)	ogm[GRCh38] t(4;7)(p16.1;q11.22)(7498374;71321506)
	t(1;7)	gpm[GRCh38] t(1;7)(q42.13;p22.1)(229625001;4780001)	ogm[GRCh38] t(1;7)(q42.13;p22.1)(229627661;4784641)
	t(1;7)	gpm[GRCh38] t(1;7)(q44;p22.1)(248170001;6085001)	ogm[GRCh38] t(1;7)(q44;p22.1)(248168445;6121028)
	t(1;7)	gpm[GRCh38] t(1;7)(q44;q11.21)(248170001;67460001)	ogm[GRCh38] t(1;7)(q44;q11.21)(248191628;67453424)
	t(7;7)	gpm[GRCh38] t(7;7)(p22.3;q11.21)(72840001;6080000)	ogm[GRCh38] t(7;7)(p22.3;q11.21)(72067101;6085026)
	t(7;7)	gpm[GRCh38] t(7;7)(p22.1;q11.23)(72840001;6350000)	ogm[GRCh38] t(7;7)(p22.1;q11.23)(72841562;6483388)

*GPM (Hi-C)*, genomic proximity mapping (GPM) (aka high-throughput chromosome conformation capture sequencing [Hi-C]); *OGM*, optical genome mapping.

No clinically reportable CNVs were identified in the proband’s mother by OGM, consistent with the findings by CMA. The translocation events in the proband’s mother are more complicated. Nine rearrangements were identified in total, including 7 interchromosomal rearrangements and 2 intrachromosomal rearrangements (Figure 3C). All 5 interchromosomal rearrangements found in the proband are also present in her mother, but the latter has 2 additional rearrangements between 1q44 and 11q25, and between 1q44 and 7p22 (Figure 3D). The intrachromosomal rearrangements were only found in the proband’s mother and formed within chromosome 7 (between 7p22 and 7q11) (Figure 3D). These results suggest that the majority of the differences between the proband and her mother are associated with 1q44, 11q25, and 7p22, supporting our previous FISH findings.

**Figure 4:**
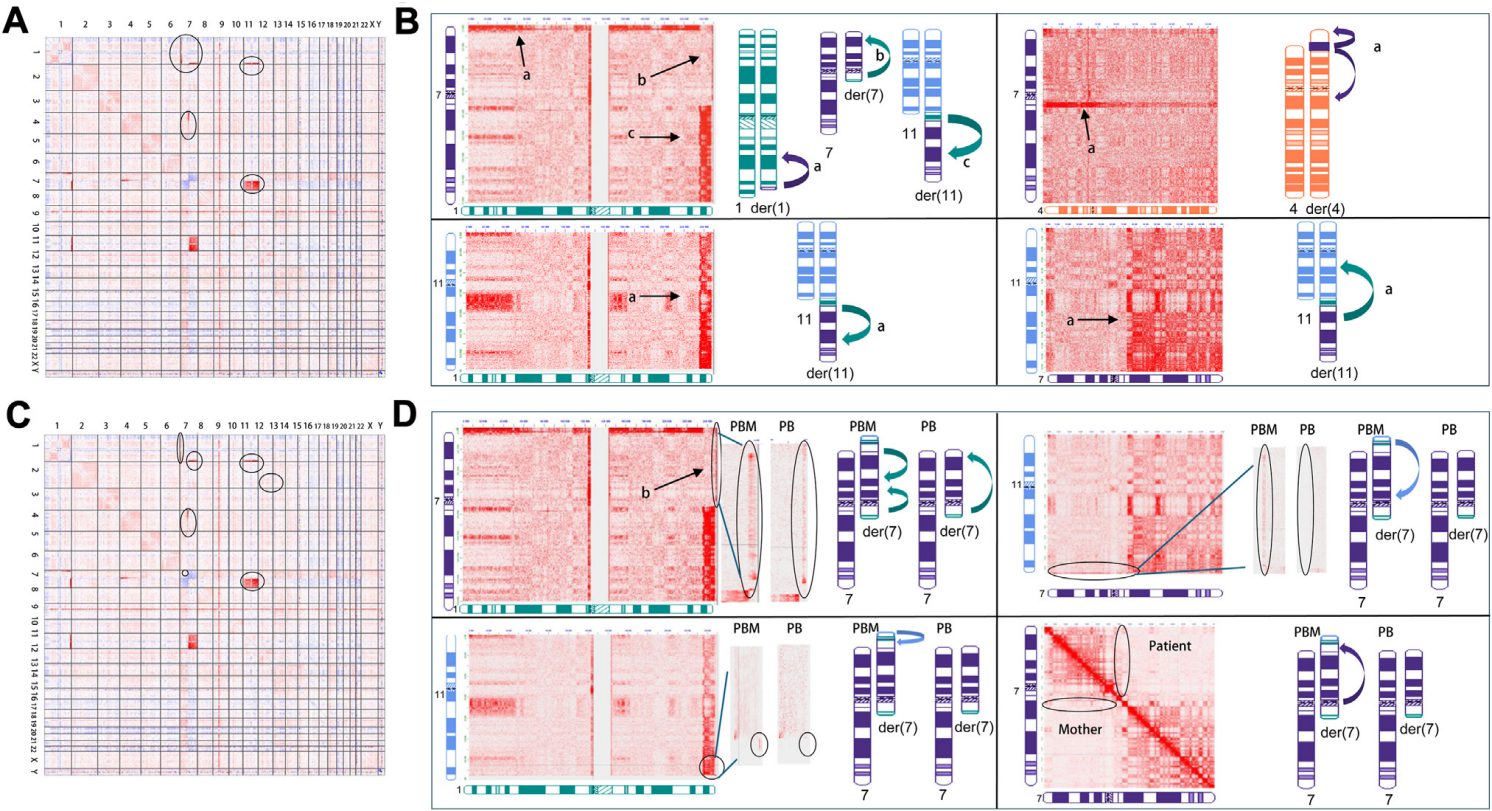
**GPM (Hi-C) results in the proband (A and B) and her mother (C and D).** (A and C) Genome-wide differential Hi-C heatmaps represent the complex chromosomal rearrangements in the proband and her mother when compared with a normal female control. Red color indicates gain of interactions in the sample and blue color indicates loss of interactions in the sample. Chromosome by chromosome view of Hi-C heatmaps in the proband (B) and her mother (D): 4 pairs of inter-chromosomal interactions were shown for the proband (B), including chromosome 1 with 7, chromosome 1 with 11, chromosome 4 with 7, and chromosome 7 with 11; 3 pairs of interchromosomal interactions and one pair of intrachromosomal interactions specifically observed in the proband’s mother (D). Each left panel in (B) and (D) shows the original heatmap, and the right panel shows the representative ideogram of the translocation. PB, proband; PBM, proband’s mother.

GPM (Hi-C) verified the proband’s CNVs as identified by CMA and OGM (Table 1). GPM (Hi-C) also successfully detected the translocations between chromosome 1, 7, and 11 and the novel insertion between chromosome 4 and chromosome 7 at a high resolution (Figure 4A). In the proband, all 5 rearrangements were detected by GPM (Hi-C) (Figure 4B), and the coordinates are listed in Table 2. To schematically represent identified variants, each translocation observed on the heatmap is presented by an ideogram. For example, we observed abnormal stripes of pairwise sequence interactions (as indicated by a, b, and c) on the contact map between chromosome 1 and chromosome 7 (Figure 4B, top left). The stripe a of signal indicated by an arrow shows an interchromosomal interaction between chromosome 7 (0-8 Mb) and chromosome 1 (0-235 Mb), identifying a translocation of 7p22 to 1q42. Similarly, from stripes b and c, we can infer 2 additional rearrangements between fragments of chromosome 1 and chromosome 7. Of note, only the rearrangements represented by the relevant chromosomes are taken into consideration to construct the ideogram up until this point of the analysis. Overall, these rearrangements are concordant with the aberrations we observed by OGM.

We also examined the CCR in the proband’s mother by GPM (Hi-C). No CNVs were identified, consistent with the findings by OGM and CMA. In addition, the genome-wide GPM (Hi-C) interaction heatmap looks very similar to the proband, showing all the rearrangements observed in the proband (Figure 4C). The subtle differences in the proband’s mother’s specific rearrangements became more evident when we examined the contact heatmap chromosome by chromosome (Figure 4D). We examined in detail the translocation between chromosome 1q44 to chromosome 7q22 (signal stripe b in the proband. When we zoomed in, we observed clear differences in the contact pattern in the proband’s mother as shown in Figure 4D. The variant is composed of 3 segments, including an interaction between 1q44 (248-249 Mb) and chromosome 7 (67-5 Mb), an interaction between 1q44 (247-248 Mb) and chromosome 7 (5-67 Mb, different orientation), and a small block between 1q44 (247-250 Mb) and chromosome 7 (72-73 Mb) (Supplemental Figure 2). With this information, we can infer complicated translocations as shown in the ideogram in Figure 4D. The additional translocations identified in the proband’s mother by OGM, including interactions between 1q44 and 7p22, 1q44 and 11q25, and 7p22 and 7q11, were also evident by GPM (Hi-C) (Figure 4D).

**Figure 5:**
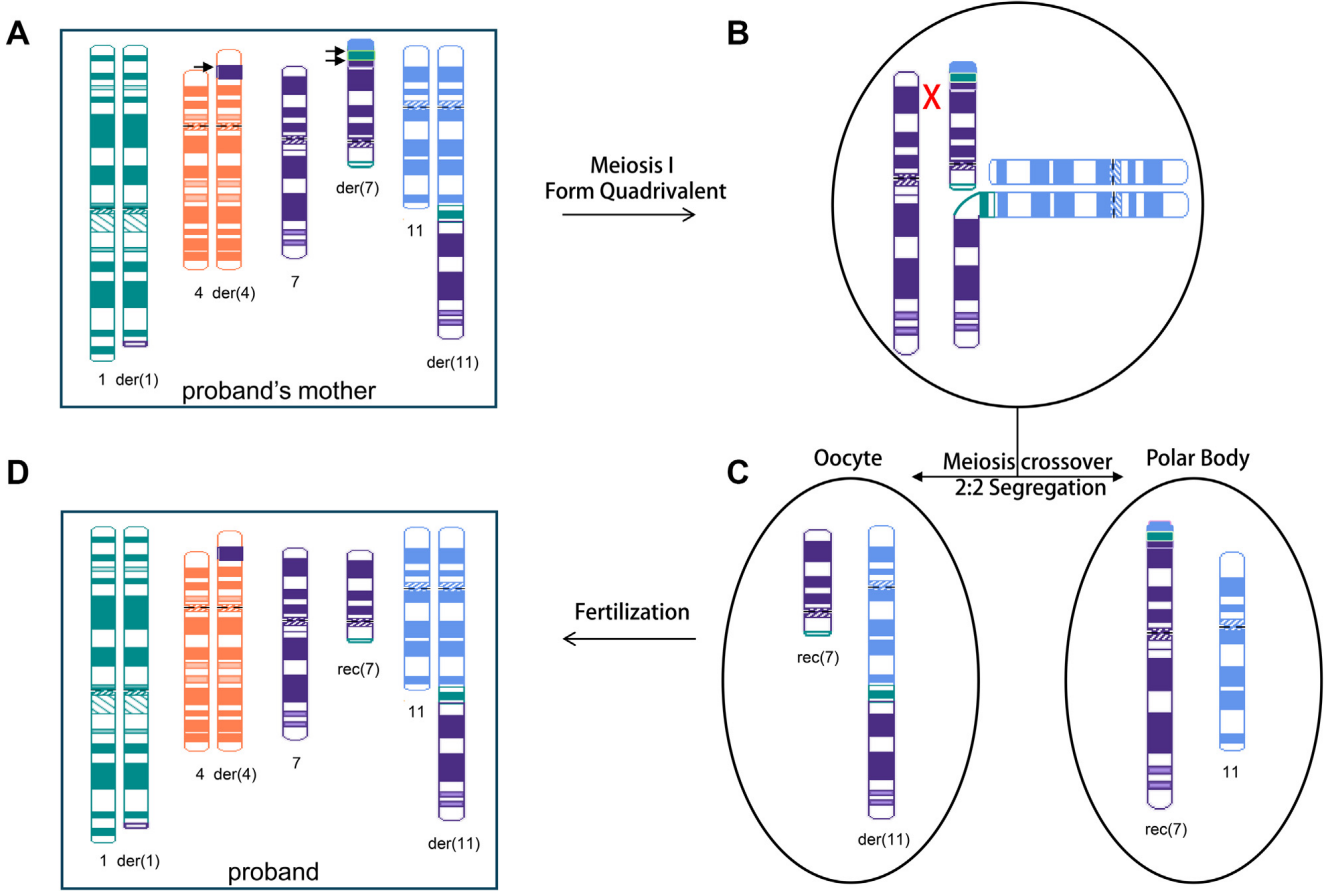
**Proposed model of complex chromosomal rearrangement (CCR) transmission from proband’s mother to the proband, resulting in unbalanced chromosomal regions.** Postulated CCRs in the proband’s mother (A) and the proband (D) include chromosome 1, 4, 7, and 11. Although derivative chromosome 7p in the proband’s mother (A) comprises segments of 1q, 11q, and 7q, recombinant chromosome 7 rec(7) in the proband contains an intact 7p sequence. Hypothesized meiotic recombination event in the proband’s mother, in which 3-way CCR heterozygote forms a quadrivalent structure (B). Crossover occurs between homologous 7p and derivative 7p and followed by 2:2 alternative segregation (C). The 3 arrows in (A) indicate the novel structural variants identified by GPM and OGM.

The long-range interactions between the translocated segments and distant neighboring genomic regions were detected by GPM (Hi-C), in addition to the interactions directly generated by the conjugated segments, provide us with supporting information related to the chromosomal rearrangement structure. For example, the proband’s mother had 2 stripes of contacts between 1q44 and chromosome 7. One of the stripes showed a more intense signal toward 7p, indicating that this fragment is translocated to 7p. The second interaction showed more intense signal proximal to 7cen, indicating that this fragment is translocated closer to 7cen. With these considerations, we were able to reconstruct the CCRs in the proband and her mother (Ideogram Figure 5A and B). OGM has shown promise in reconstructing complex CCRs in some cases, although its effectiveness decreases as the number of breakpoints increases, especially when closely positioned breakpoints involve multiple translocations. GPM (Hi-C), which measures long-range interactions, provides a comprehensive view of rearranged fragment locations, aiding in the reconstruction of intricate CCRs by clarifying the spatial arrangement of these fragments. Notably, both OGM andGPM (Hi-C) are methods to measure genomic segments, and they are not capable of mapping the whole genome continuously and directly. Because of the complexity of these rearrangements and the limitation of the detection methods, we cannot rule out other possible rearrangements.

Overall, we were able to reconstruct the complicated CCRs for both the proband and her mother. We also confirmed that both OGM and GPM (Hi-C) can facilitate robust detection of cryptic balanced and unbalanced translocations in clinical practice with a high degree of accuracy.

### Effect of the CCR on gene expression

To identify dysregulated genes and pathways associated with the proband’s clinical presentation, we performed transcriptome analysis by mRNA-seq of the proband’s and her mother’s peripheral blood samples. We also compared expression profiles in the proband and her mother with public mRNA-seq data sets from peripheral blood samples of age-matched control females.

We first examined the expression of genes spanning the breakpoints of the CCRs in the proband, including *ARID4B*, *GLB1L2*, *SORCS2*, *URB2*, and *AP5Z1*. RNA-seq revealed a hybrid transcription product of *ARID4B* and *GLB1L2*, confirming the t(1;11) translocation revealed by other methods (Supplemental Figure 3). The same transcription product was found in the proband’s mother, with a similar level of expression, and is absent in healthy controls. *SORCS2*, *URB2*, and *AP5Z1* all have a median expression less than 1 transcript per million in the proband, her mother, and in healthy control data sets from Gene Expression Omnibus and Genotype-Tissue Expression whole blood database,^18^^,^^19^ indicating that they are not expressed in whole blood (Supplemental Table 1). We next examined the expression of genes fully contained within the CNVs. There are 156 such genes, including 71 coding genes, 72 noncoding genes, and 13 pseudo genes. Of the 71 coding genes, 23 are in the olfactory gene family (OR genes). None of these 156 genes shows dosage sensitivity according to ClinGen^20^ (Supplemental Table 1). Differential gene expression analysis showed that only *TRIM58* (1q44) varies significantly in the proband compared with her mother and the public data sets (Supplemental Figure 4A). The proband’s *TRIM58* expression is 3.15-fold lower than her mother’s and 2.68-fold lower than normal female controls, both with FDR < 0.01. The TRIM58 gene is exclusively expressed in late-stage erythroblasts and involved in erythrocyte development. Taken together, the differential gene expression analysis using RNA-seq with the blood samples suggests that most genes spanning the CCR breakpoints or within the CNVs are unlikely to have caused the proband’s phenotype.

**Figure 6:**
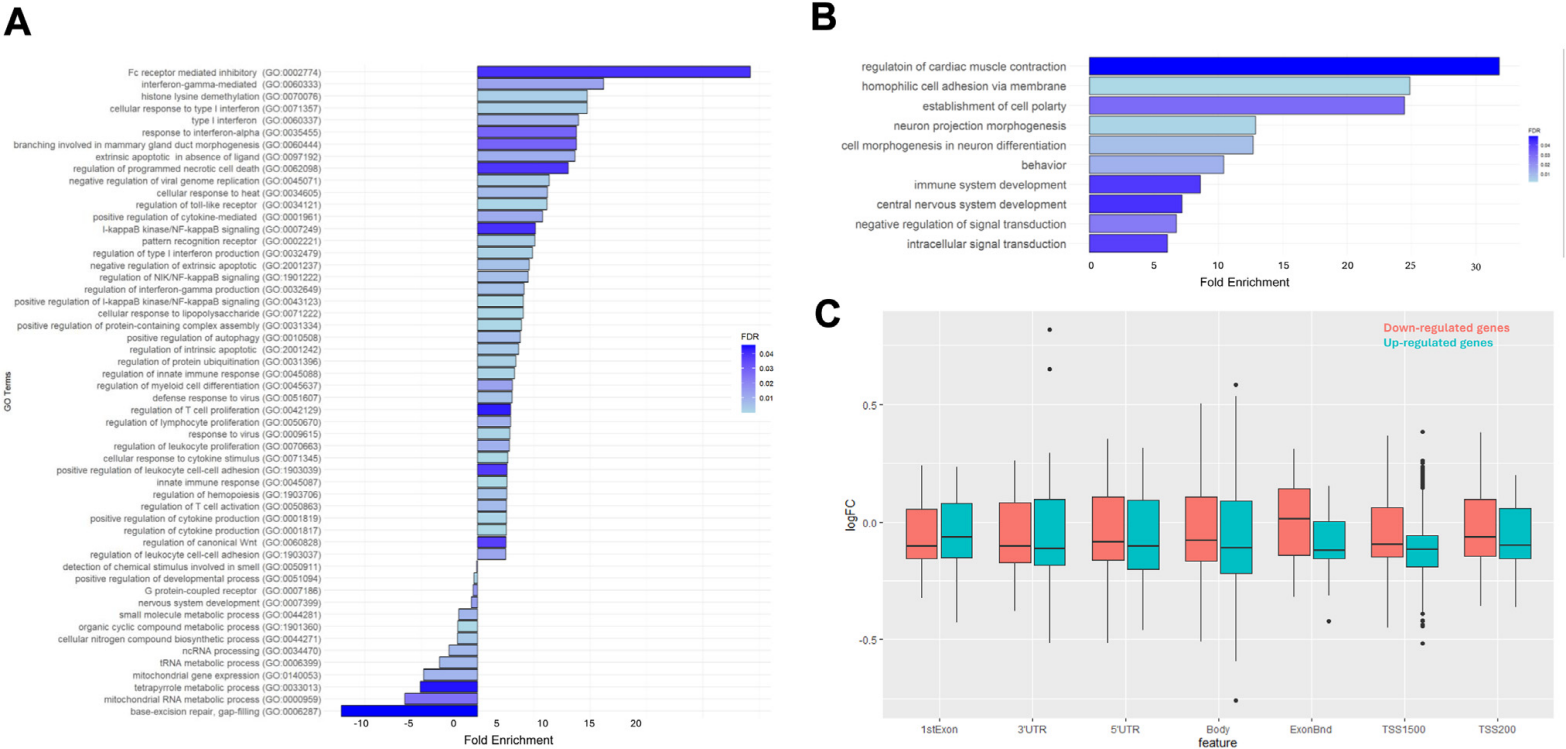
**Impact of the complex chromosomal rearrangements on gene expression and DNA methylation.** Differential expression analysis of genes in copy-number variant regions by comparing the proband with normal female controls and proband’s mother (orange and blue dots, respectively). A. RNA sequencing identified pathways affected in the proband. Upregulated genes in immune response and downregulated genes in neuronal development. B. Functional pathways affected identified by top genes enriched by significant CpGs. C. Correlation of the DNA methylation profile with the RNA expression profile. Red color bar indicates the methylation change of downregulated genes and blue color bar indicates the methylation change of upregulated genes. FC, fold change; GO, Gene Ontology; ncRNA, non-coding RNA; tRNA, transfer RNA; UTR, untranslated region.

To address the effect of the CCR on global gene expression, we identified DEGs in the proband compared with her mother and age-matched controls. We identified 510 and 757 genes that are up- and downregulated, respectively (Supplemental Table 2). These genes are uniformly located across chromosomes, without significant enrichment on any specific chromosome (Supplemental Figure 4B). GO analysis was performed for upregulated and downregulated DEGs separately, which has been shown to be more powerful than using all DEGs together to identify disease-associated pathways (Figure 6A). Upregulated DEGs are highly enriched in immune response processes, including Fc receptor-mediated inhibitory signaling pathway, interferon-gamma-mediated signaling pathway and cellular response to type I interferon pathway. These pathways could be associated with the proband’s autoimmune disorders, including SLE with Sjogren syndrome, suspected lupus nephritis, and ITP. In contrast, downregulated genes are enriched in general biological processes, including base-excision repair and mitochondrial RNA metabolic process.

### Effect of the CCR on the epigenetic landscape

The epigenetic landscape encompasses multiple key features, including methylation profiles and chromosomal organization, which together contribute to the regulation of gene expression and cellular identity. We first analyzed the methylation profiles of the proband and her mother. The top 5000 CpGs with significantly differential methylation status in the proband were identified and these CpGs are distributed evenly across the genome (Supplemental Figure 4C). The chromosomes involved in the CCRs (chromosomes 1, 4, 7, and 11) are not enriched for differential CpGs, suggesting that the CCR does not lead to major alterations in the proband’s methylation landscape. GO analysis was performed for the top 70 genes associated with these CpG sites (Figure 6B) and showed that the proband’s top genes with differential methylation status are highly enriched in neuronal developmental processes relevant to the proband’s developmental delay, including neuron projection morphogenesis, neuron differentiation, and establishment of cell polarity.

We further correlated the DNA methylation profile with the RNA expression profile. The median methylation level is increased for the TSS (transcriptional start site) regions of downregulated genes and decreased for the upregulated genes, consistent with hypermethylation of downregulated genes and hypomethylation for upregulated genes (Figure 6C). In contrast, the proband’s differential methylation analysis identified 2142 hypomethylated probes and 235 hypermethylated probes, corresponding to the hypomethylation of TSS regions of 1239 genes and hypermethylation of TSS regions of 126 genes. We did not observe significant changes in expression of these genes, likely because most of these genes are not expressed in blood cells.

Chromosomal organization is another key aspect of the epigenetic landscape. We first assessed the changes of the proband’s overall chromosomal organization. To do this, we computed the similarities between the genome-wide Hi-C contact matrix of the proband, her mother and a healthy control with HiCRep,^21^ a tool that can provide a statistical evaluation of the Hi-C interaction matrix (Supplemental Figure 5A). The Hi-C contact matrix in the proband showed high correlations (>0.89) with both the healthy controls and the proband’s mother. Chromosomes involved in the CCR (chromosomes 1, 4, 7, and 11) present with similar correlation compared with other chromosomes, suggesting that the translocations do not lead to major alterations in the proband’s chromosomal organizations.

Having established the overall correlation between the Hi-C interaction matrices of the 2 samples, we next conducted a detailed analysis of the TADs and A/B compartments to investigate the similarities and differences in chromatin organization between the samples. TADs are regions of the genome with high intradomain chromatin interactions and low interdomain interactions, which are thought to play a role in gene regulation and chromatin organization. A/B compartments refer to large-scale chromatin domains that have distinct epigenetic and transcriptional properties, with A compartments enriched for active chromatin marks and highly transcribed genes, and B compartments enriched for repressive marks and lowly expressed genes. We examined the A/B compartments and TAD organizations. No A/B compartment changes are observed except the regions around translocation breakpoints (Supplemental Figure 5B). The TAD organizations are also maintained in the context of complicated translocations in both the proband and proband’s mother (Supplemental Figure 5C).

## Discussion

CCRs are rare, with several hundred cases involving 3 or more chromosomes reported, most of which are de novo.^2^^,^^11^ Retrospective analyses estimate the occurrence of CCRs at approximately 0.5% in newborns.^22^ CCRs can present as unbalanced, apparently balanced by conventional banding techniques, or truly balanced configurations. However, the prevalence of balanced CCRs in the population may be underestimated because they often go undetected by traditional methods, especially when they do not produce a phenotypic effect. This underestimation is evident in the family we present, in which the mother, displaying no apparent abnormal phenotype, was identified with CCRs only after her daughter’s diagnosis.

Recognizing individuals with CCRs is clinically essential because they face increased risks of miscarriage and bearing children with unbalanced karyotypes. However, accurately identifying CCRs can be challenging with conventional cytogenetic methods. In our study, both the mother and daughter exhibited apparently balanced CCRs on banding techniques and yet showed distinct molecular profiles. This case highlights the high utility of novel molecular cytogenetic technologies, such as OGM and GPM (Hi-C), which provide a clearer, more precise characterization of CCRs that conventional methods cannot achieve.

Assessing CCRs and their potential phenotypic impacts remains a significant challenge in clinical genetics. Developmental delay is among the most common clinical indications in individuals with unbalanced CCRs, often resulting from pathogenic CNVs.^23^^,^^24^ CMA can efficiently identify CNVs and has become the first-tier test for people with unexplained developmental delay. For example, a study of 329 people with intellectual disability confirms that causative CNVs are frequently found, even in cases of mild intellectual disability.^25^ The proband in our study is a 31-year-old female with delayed speech, cognitive impairment, and autoimmune disorders. CMA identified a 768 kb deletion of 1q44 that affects *NLRP3*, *GCSAML*, and *OR*, a 788 kb deletion of 7q11.22 to 7q11.23 that affects *CALN1* and *TYW1B*, a 709 kb deletion of 11q25 that affects *GLB1L2* and *B3GAT1*, and a 4.7 Mb duplication of 7p22.3 to 7p22.1, involving 37 genes. The CNVs do not overlap any known dosage sensitive genes or regions, and the classification of each of these regions is uncertain per ACMG interpretation standards.^26^ Thus, these CNVs may be associated with the proband’s phenotype, but more evidence is needed to determine if the chromosomal rearrangements are related to the underlying molecular mechanism the proband’s condition.

Further investigation revealed the inherited nature of the proband’s CCR by both conventional and novel cytogenetic methods. Conventional karyotype analysis revealed that the proband and her mother had nearly indistinguishable chromosomal abnormalities, characterized by 3 translocations involving 3 different chromosomes. Additional FISH analysis successfully identified specific chromosomal rearrangements localized to chromosomal 7p in the proband and proband’s mother but did not precisely localize the breakpoints. CMA effectively identified the coordinates of the breakpoints of the imbalanced rearranged chromosomal segments. Still without further details, the chromosomal rearrangements could not be fully resolved in this family. Both OGM and GPM (Hi-C) identified a novel translocation between chromosomal 4 and chromosomal 7 in the proband and her mother. OGM and GPM (Hi-C) also uncovered a set of unique rearrangements only carried by the proband’s mother, including an intra-chromosomal translocation within chromosome 7, an additional translocation between chromosome 1 and chromosome 11, and an additional translocation between chromosome 1 and chromosome 7. These observations, especially the translocations exclusively detected in the proband’s mother, led us to suspect a more complex rearrangement exists in proband’s mother and that such rearrangement spans chromosomes 1, 4, 7, and 11. Moreover, GPM (Hi-C) was able to show relative position between translocated segments; therefore, we were able to extrapolate that the unique sets of rearrangement events carried by the proband’s mother are sequentially located on the chromosome 7p arm. For example, the Hi-C heatmap shows 2 stripes of sequence interaction indicating a translocation between chromosomes 1 and 7, revealing increased interaction between regions 1q44 and 7p22q11. The 2 stripes, labeled A and B, differ in orientation. This suggests that the terminal fragment (278.1-248.9 Mb) of 1q44 is located at 7q11, whereas the interstitial fragment (247.4-248.1 Mb) of 1q44 is closer to 7p22. (Figure 4, Supplemental Figure 2). Overall, we reconstructed the CCR in the proband and her mother. We propose a model to explain how the CCR was passed from the proband’s mother to the proband, leading to multiple unbalanced chromosomal regions (Figure 5). We suspect that there was a recombination event between the normal and derivative chromosomes 7 at 7p during meiosis 1 pairing in the proband’s mother involving chromosome 7, the derivative chromosome 7, chromosome 11, and the derivative chromosome 11. During meiosis I, the 3-way CCR heterozygote came together to form a quadrivalent configuration (Figure 5B). Meiotic crossover occurred between homologous chromosome 7p and derivative chromosome 7p followed by 2:2 alternative segregation (Figure 5C). Thus, recombinant chromosome 7 in the proband (Figure 5D) has a normal chromosome 7p, whereas the der(7) in her mother (Figure 5A) has a 7p composed of part of 1q, 11q, and 7q. This crossover would also explain the CNVs observed in the proband, whereas her mother is balanced.

In addition, we investigated the genetic cause of the proband’s clinical presentations. When investigating the genetic basis of abnormalities in individuals with unbalanced CCRs, the approach typically focuses on evaluating genes near the breakpoints and examining any resulting CNVs. With OGM and GPM (Hi-C), we identified a total of 8 breakpoints and 4 regions with CNVs in the proband. Interestingly, there are 13 breakpoints in total in the proband’s mother, including the 8 breakpoints in the daughter and an additional 5 breakpoints found only in the proband’s mother, with no CNVs. None of these genes around the breakpoints are known morbid genes by Online Mendelian Inheritance in Man. In addition, considering that the proband’s mother is phenotypically unremarkable, we suspect that the breakpoints are unlikely to account for the proband’s phenotype. Instead, the CNVs and their interactions with the global transcriptome and epigenetic landscape are likely to be responsible. CMA identified in the proband do not overlap any known dosage sensitive genes or regions, and the classification of each of these regions is uncertain per ACMG interpretation standards. Thus, we performed transcriptome and methylome analysis in the proband. The expression and methylation profiles of the genes directly located within the CNV or spanning the breakpoints appeared unaffected in the proband. In contrast, we identified functional pathways potentially associated with the proband’s phenotype using the global DE and methylation profiles, suggesting that it is the disruption of cellular networks that leads to the proband’s findings. For example, we identified upregulated genes highly enriched in pathways associated with immune response processes, including Fc receptor-mediated inhibitory signaling pathway, interferon-gamma-mediated signaling pathway and cellular response to type I interferon pathway. These pathways could be associated with the proband’s autoimmune disorders, including SLE with Sjogren syndrome, suspected lupus nephritis, and ITP. Similarly, the proband’s top genes with differential methylation status are highly enriched in neuronal developmental processes relevant to the proband’s developmental delay, including neuron projection morphogenesis, neuron differentiation, and establishment of cell polarity. In addition, the proband’s transcriptome and methylome profiles correlated with the TSS regions hypermethylated for downregulated genes and hypomethylated for the upregulated genes. Although the transcriptome analysis identified a set of genes potentially relevant to the proband’s immune deficiency, the methylome analysis identified a set of genes potentially relevant to the proband’s developmental delay. Although the DEGs identified by RNA-seq did not overlap with the differentially methylated genes identified by DNA methylation array, it is known that changes in gene expression may not always correlate directly with DNA methylation status because other regulatory elements can modulate gene expression independently of methylation.

Although our study is limited to a single family and lacks broad generalizability, it highlights the complex, multifaceted role of chromosomal rearrangements in shaping phenotype. Rather than identifying a single causative gene, we observed widespread transcriptional and structural shifts, suggesting a network of genomic alterations that may drive the observed phenotype. The small sample size and use of peripheral blood rather than directly relevant tissues—such as neural tissue for developmental issues or immune cells for autoimmune symptoms—do restrict the depth of our insights. Moreover, the complexity of the CCRs, with numerous chromosomal breakpoints and rearrangements, complicates pinpointing specific causative regions. These findings emphasize the challenges in interpreting chromosomal rearrangements and suggest that their effects may reach beyond isolated gene disruptions. Future studies with larger cohorts and analyses of relevant tissues are essential to validate and expand on these findings.

In summary, this study demonstrates the power of advanced cytogenetic methods, including OGM and GPM (Hi-C), in resolving CCRs and their pathophysiologic consequences. Conventional cytogenetic methods are routinely applied in the cytogenetics lab. Although these are powerful diagnostic tools, they also have limitations, particularly in defining CCRs with limited resolution, as seen with this case. This study showcases the unique strengths and limitations of each method (Table 3) and provides an example of how the complementary approaches of OGM, high-throughput GPM (Hi-C), and conventional cytogenetics provide a comprehensive characterization of complex SVs. Our approach also highlights how RNA-seq and methylome analyses can further inform our understanding of the molecular underpinnings of phenotypes of patients with CCRs.

**Table 3:** Strength and limitation of FISH, karyotype, SNP CMA, GPM (Hi-C), and OGM in the detection of CNVs and SVs, including CCRs

	FISH	Karyotype	CMA	GPM (Hi-C)	OGM
Region	targeted region	genome wide	genome wide	genome wide	genome wide
Resolution	100 kb (medium)	5 Mb (low)	40 kb (high)	1 kb ∼ 50 kb (high)	500 bp ∼ 50 kb (high)
Detection of CNV	Yes	Yes	Yes	Yes	Yes
Unbalanced SV	Yes	Yes	Yes	Yes	Yes
Balanced SV	Yes	Yes	No	Yes	Yes
Reconstruction of complex CCR	No	Yes	No	Yes^a^	Yes^a^

*CCR*, complex chromosomal rearrangement; *CMA*, chromosomal microarray analysis; *CNV*, copy-number variant; *FISH*, fluorescence in situ hybridization; *GPM (Hi-C)*, high-throughput chromosome conformation capture sequencing (Hi-C; aka genomic proximity mapping [GPM]); *OGM*, optical genome mapping; *SNP*, single-nucleotide polymorphism; *SV*, structural variant.

a OGM and Hi-C can effectively reconstruct complex CCRs in certain cases and has been reported. OGM offers a straightforward visualization. Reconstruction becomes challenging as breakpoints increase, particularly if closely located breakpoints involve different translocations. Hi-C requires a learning curve but aids complex reconstructions by providing an overview of rearranged fragment locations through long-range interactions.

## Data Availability

Anonymized data can be made available upon request and with appropriate agreements and human research ethics committee approval.

## Conflict of Interest

Stephen M. Eacker is an employee of Phase Genomics, Inc, the developer of the GPM technology. All other authors declare no conflicts of interest.

## Additional Information

Suplementary Figures 1-5

## References

[1] Patsalis P.C. (2007). Complex chromosomal rearrangements. Genet Couns.

[2] Pellestor F., Anahory T., Lefort G. (2011). Complex chromosomal rearrangements: origin and meiotic behavior. Hum Reprod Update.

[3] Batista D.A., Pai G.S., Stetten G. (1994). Molecular analysis of a complex chromosomal rearrangement and a review of familial cases. Am J Med Genet.

[4] Zepeda-Mendoza C.J., Morton C.C. (2019). The iceberg under water: unexplored complexity of chromoanagenesis in congenital disorders. Am J Hum Genet.

[5] de Vree P.J., Simon M.E., van Dooren M.F. (2009). Application of molecular cytogenetic techniques to clarify apparently balanced complex chromosomal rearrangements in two patients with an abnormal phenotype: case report. Mol Cytogenet.

[6] Madan K., Nieuwint A.W., van Bever Y. (1997). Recombination in a balanced complex translocation of a mother leading to a balanced reciprocal translocation in the child. Review of 60 cases of balanced complex translocations. Hum Genet.

[7] Bonaglia M.C., Giorda R., Borgatti R. (2001). Disruption of the ProSAP2 gene in a t(12;22)(q24.1;q13.3) is associated with the 22q13.3 deletion syndrome. Am J Hum Genet.

[8] Puissant H., Azoulay M., Serre J.L., Piet L., Junien C. (1988). Molecular analysis of a reciprocal translocation t (5; 11)(q11; p13) in a WAGR patient. Hum Genet.

[9] Madan K. (2012). Balanced complex chromosome rearrangements: reproductive aspects. A review. Am J Med Genet A.

[10] Poot M., Haaf T. (2015). Mechanisms of origin, phenotypic effects and diagnostic implications of complex chromosome rearrangements. Mol Syndromol.

[11] Sinkar P., Devi S.R. (2020). Complex chromosomal rearrangement: a case report to emphasize the need for parental karyotyping and genetic counseling. J Hum Reprod Sci.

[12] Dremsek P., Schwarz T., Weil B., Malashka A., Laccone F., Neesen J. (2021). Optical genome mapping in routine human genetic diagnostics-its advantages and limitations. Genes (Basel).

[13] Harewood L., Kishore K., Eldridge M.D. (2017). Hi-C as a tool for precise detection and characterisation of chromosomal rearrangements and copy number variation in human tumours. Genome Biol.

[14] Reymond A., Henrichsen C.N., Harewood L., Merla G. (2007). Side effects of genome structural changes. Curr Opin Genet Dev.

[15] Henrichsen C.N., Chaignat E., Reymond A. (2009). Copy number variants, diseases and gene expression. Hum Mol Genet.

[16] Henrichsen C.N., Vinckenbosch N., Zöllner S. (2009). Segmental copy number variation shapes tissue transcriptomes. Nat Genet.

[17] Chakraborty A., Ay F. (2018). Identification of copy number variations and translocations in cancer cells from Hi-C data. Bioinformatics.

[18] Edgar R., Domrachev M., Lash A.E. (2002). Gene Expression Omnibus: NCBI gene expression and hybridization array data repository. Nucleic Acids Res.

[19] GTEx Consortium (2013). The Genotype-Tissue Expression (GTEx) project. Nat Genet.

[20] ClinGen Consortium (2025). The Clinical Genome Resource (ClinGen): advancing genomic knowledge through global curation. Genet Med.

[21] Lin D., Sanders J., Noble W.S. (2021). HiCRep.py: fast comparison of Hi-C contact matrices in Python. Bioinformatics.

[22] Hu L., Wei Y., Luo K. (2018). Clinical outcomes in carriers of complex chromosomal rearrangements: a retrospective analysis of comprehensive chromosome screening results in seven cases. Fertil Steril.

[23] Shoukier M., Klein N., Auber B. (2013). Array CGH in patients with developmental delay or intellectual disability: are there phenotypic clues to pathogenic copy number variants?. Clin Genet.

[24] Caramaschi E., Stanghellini I., Magini P. (2014). Predictive diagnostic value for the clinical features accompanying intellectual disability in children with pathogenic copy number variations: a multivariate analysis. Ital J Pediatr.

[25] D‘Arrigo S., Gavazzi F., Alfei E. (2016). The diagnostic yield of array comparative genomic hybridization is high regardless of severity of intellectual disability/developmental delay in children. J Child Neurol.

[26] Riggs E.R., Andersen E.F., Cherry A.M. (2020). Technical standards for the interpretation and reporting of constitutional copy-number variants: a joint consensus recommendation of the American College of Medical Genetics and Genomics (ACMG) and the Clinical Genome Resource (ClinGen). Genet Med.

