# Supplementary material for: Genetic and functional characterization of inherited complex chromosomal rearrangements in a family with multisystem anomalies
Source: Genet Med Open. 2025 Mar 11;3:103423. doi: 10.1016/j.gimo.2025.103423 (PMC12136847; doi:10.1016/j.gimo.2025.103423)

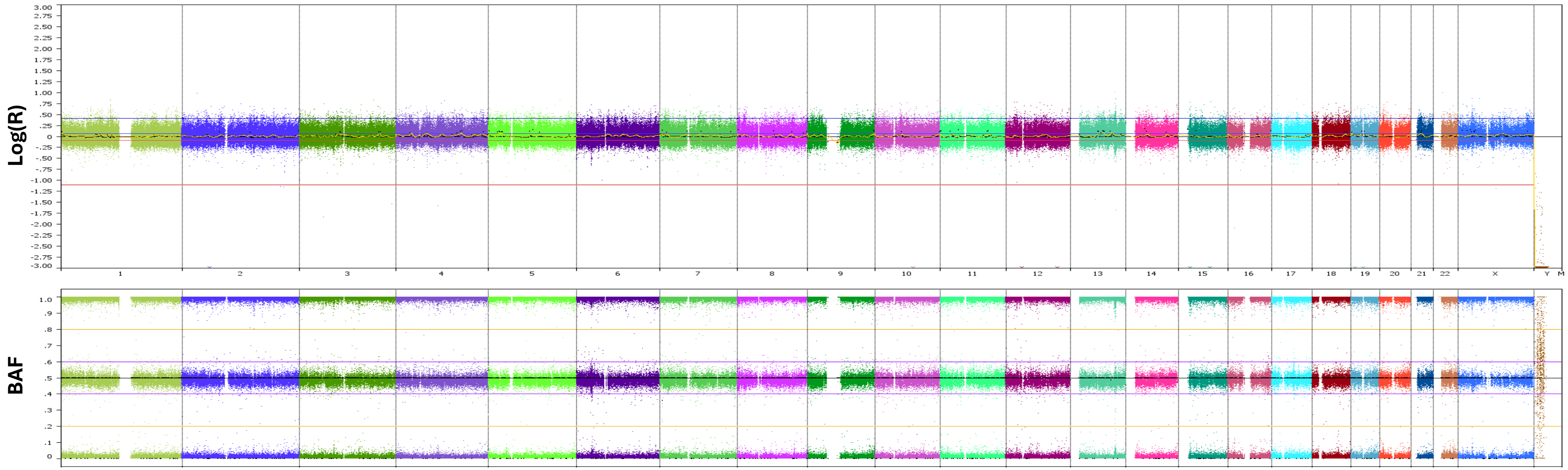

Supplementary Figure 1. Chromosomal and SNP array analysis of the proband's mother. No clinically reportable copy number change was detected in the proband's mother.

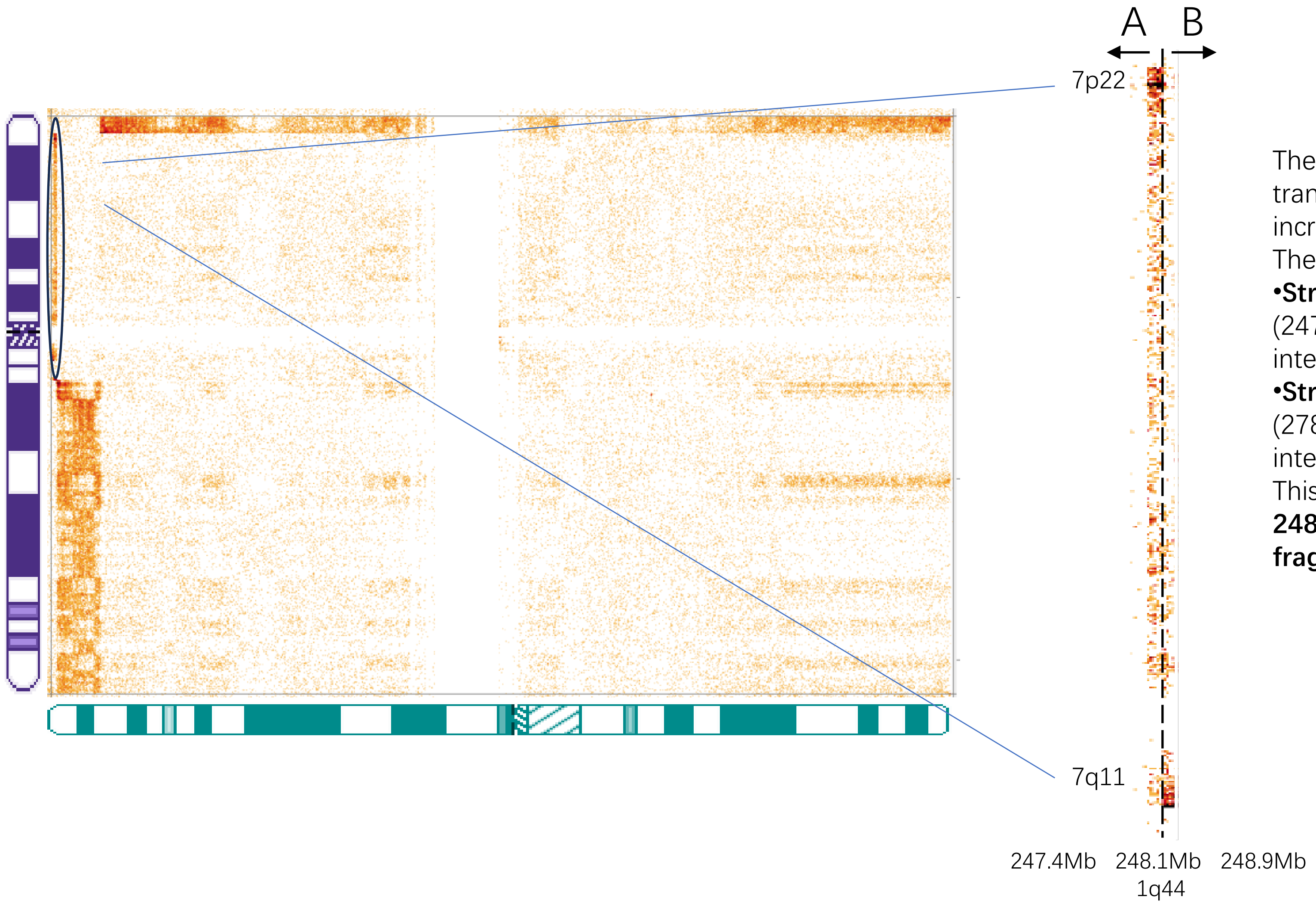

The Hi-C heatmap shows two stripes indicating a translocation between chromosomes 1 and 7, revealing increased interaction between regions 1q44 and 7p22q11.
The two stripes, labeled A and B, differ in orientation:

•**Stripe A** shows interaction between chromosome 1 (247.4Mb to 248.1Mb) and chromosome 7, with higher intensity at the 7p22 end.

•**Stripe B** shows interaction between chromosome 1 (278.1Mb to 248.9Mb) and chromosome 7, with higher intensity at the 7q11 end.

This suggests that the **terminal fragment (278.1Mb to 248.9Mb) of 1q44** is located at **7q11**, while the **interstitial fragment (247.4Mb to 248.1Mb) of 1q44** is closer to **7p22**.

Supplementary Figure 2. Example of interpreting relative locations of rearranged genomic fragments using GPM (Hi-C)

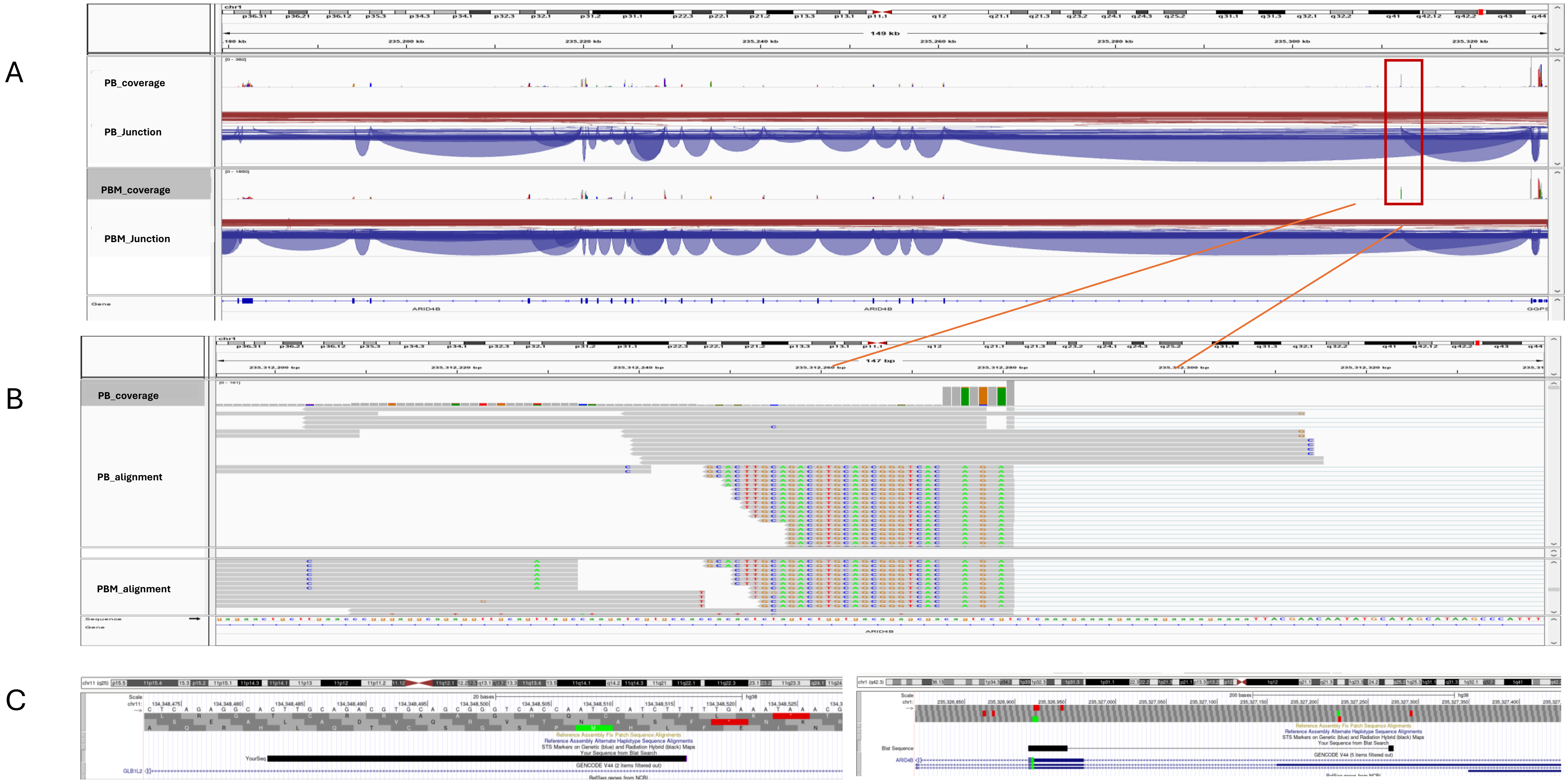

Supplementary Figure 3. RNA-seq revealed a hybrid transcription product of *ARID4B* and *GLB1L2*, confirming the t(1;11) translocation revealed by other methods. (A) Transcriptional junction detected by RNA-seq in the proband and the proband's mother. The hybrid peaks were highlighted by red square. (B) Hybrid reads alignments in the proband and the proband's mother. (C) Detailed mapping of the hybrid reads to Chr1 and Chr11 by UCSC genome browser.

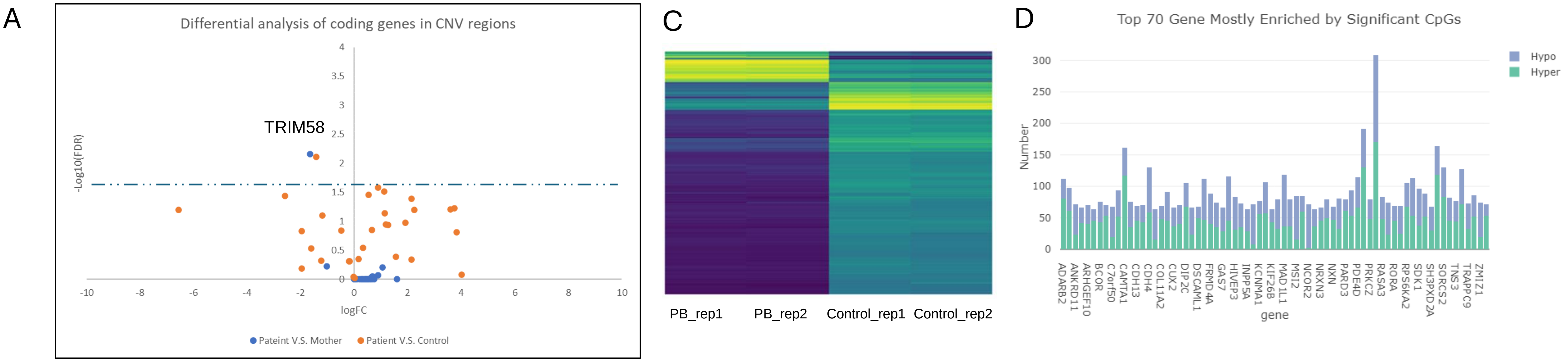

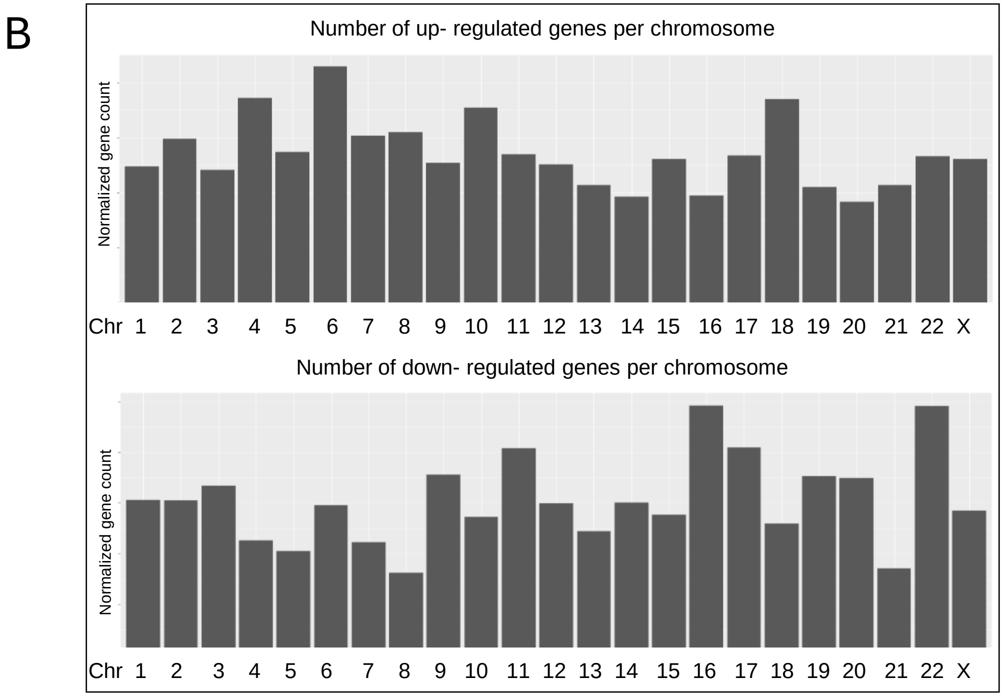

Supplementary Figure 4. A) Differential analysis of coding genes located in the CNV regions. Orange dots indicates the LogFC and –log10(FDR) comparing the genes' expression in the patient with normal female controls. Blue dots indicates the LogFC and –log10(FDR) comparing the genes' expression in the patient with her mother. Dash line indicate the cutoff of FDR<0.01. Only TRIM58 passes the cutoff line. B) Distribution of differential genes by chromosome. Number of differential genes are normalized by the total number of coding gene per chromosome. No enrichment on any chromosome is observed. C) Functional pathways affected identified by RNA-seq in the proband. Up-regulated genes are enriched in immune response associated pathways while down regulated genes are enriched in neuronal development associated pathways. C) Heatmap of the top 5000 CpG probes with significant differences. B) Differentially methylated genes identified by Illumina Infinium MethylationEPIC array in the proband. Top 70 genes enriched with significant CpGs are listed. Hypomethylated CpGs are colored by blue and hypermethylated CpGs are colored by green.

Supplementary Figure 5. Effect of the CCRs on chromosomal organization. (A) Correlation score of Hi-C interaction matrices between the proband and the proband's mother by chromosomes. (B) A/B compartments analysis in the proband and the proband's mother for chromosomes 1, 7, and 11 (red for A compartment, blue for B compartment). (C) Topologically Associating Domains (TADs) on chromosome 7 in the proband and the proband's mother.

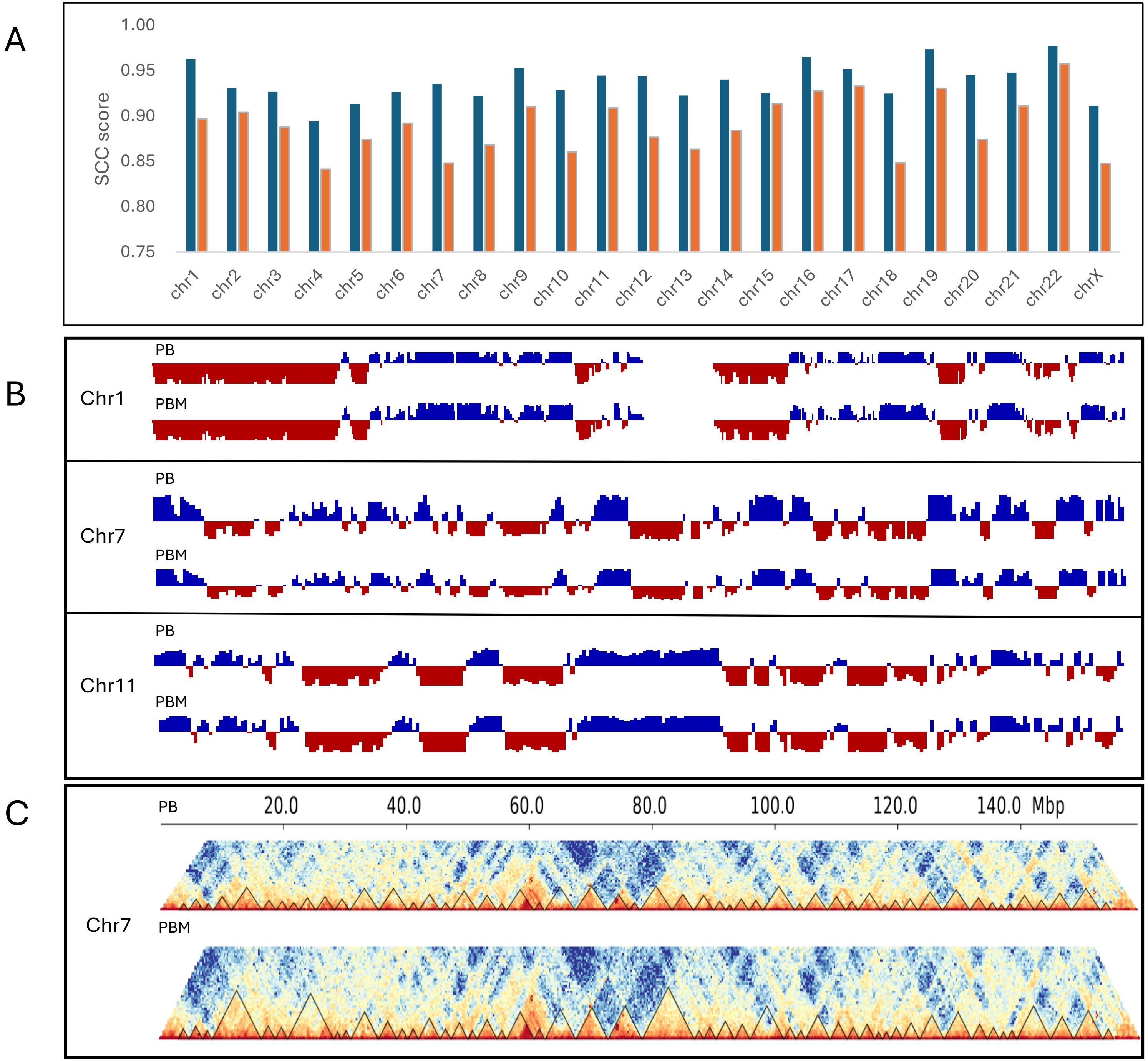

Supplement: Suplementary Figures 1-5 [file mmc1.pdf]
